# Supplementary figures and images for: Evaluation of the reliability and validity of computerized tests of attention
Source: PLoS One. 2023 Jan 27;18(1):e0281196. doi: 10.1371/journal.pone.0281196 (PMC9882756; doi:10.1371/journal.pone.0281196)

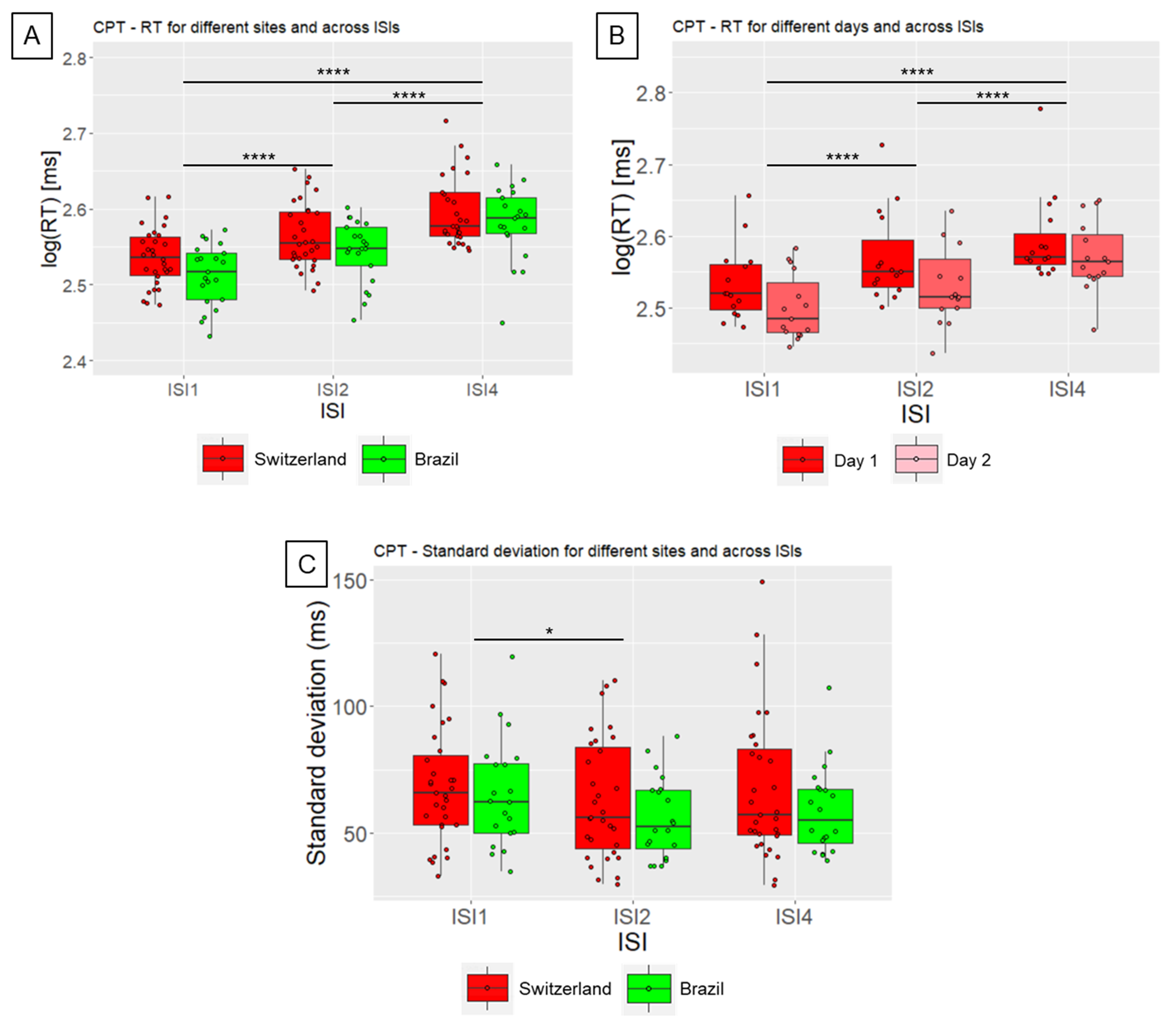

Supplement: S1 Fig — CPT reaction time (RT) as a function of inter-stimulus interval (ISI) for single (A) and double applications (B). CPT standard deviation as a function of ISI for the single application (C). Asterisks represent significant differences in post-hoc tests corrected for multiple comparisons using the Sidak method (**** p < 0.0001, * p < 0.05). ISI1, ISI2, ISI4 = inter-stimulus interval of 1, 2, and 4 s, respectively. (TIF) [file pone.0281196.s001.tif]

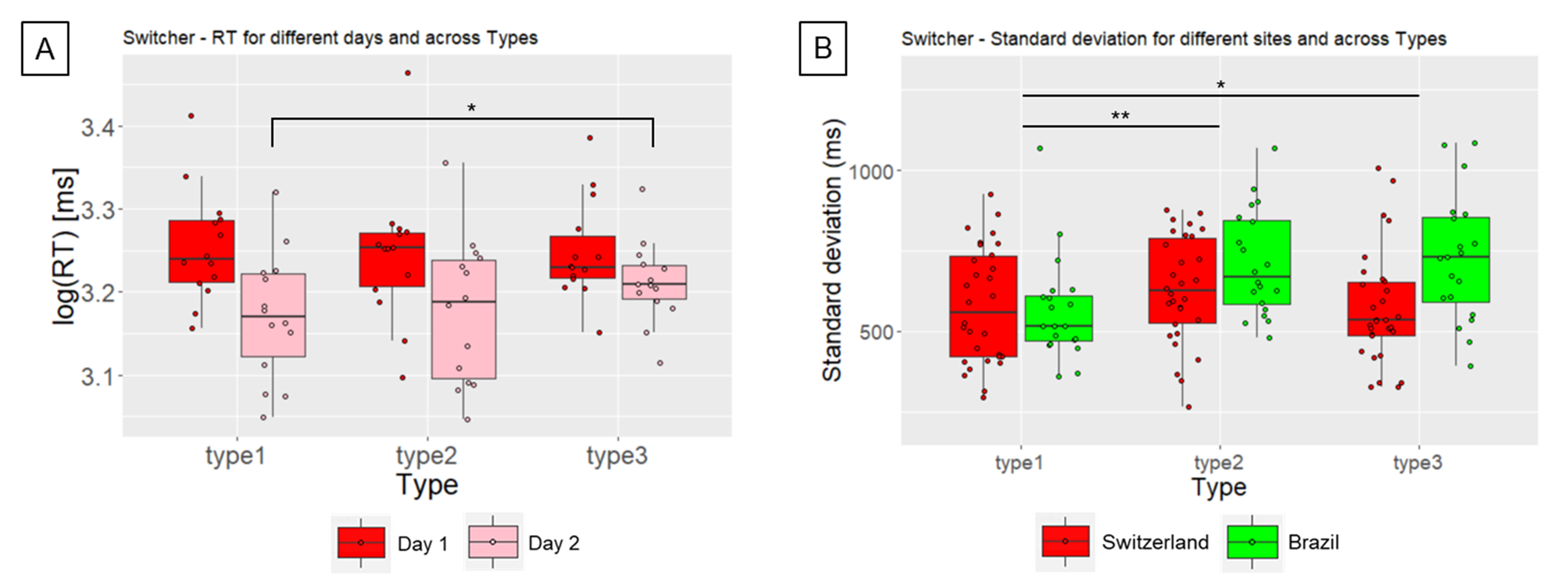

Supplement: S2 Fig — (A) Switcher task reaction time (RT) as a function of Type (Type1, Type2, Type3 = alternate switch, fixed switch, random switch, respectively) for double application. (B) Switcher task standard deviation as a function of Type for single application. Asterisks represent significant differences in post-hoc tests corrected for multiple comparisons using the Sidak method (** p < 0.01, * p < 0.05). (TIF) [file pone.0281196.s002.tif]

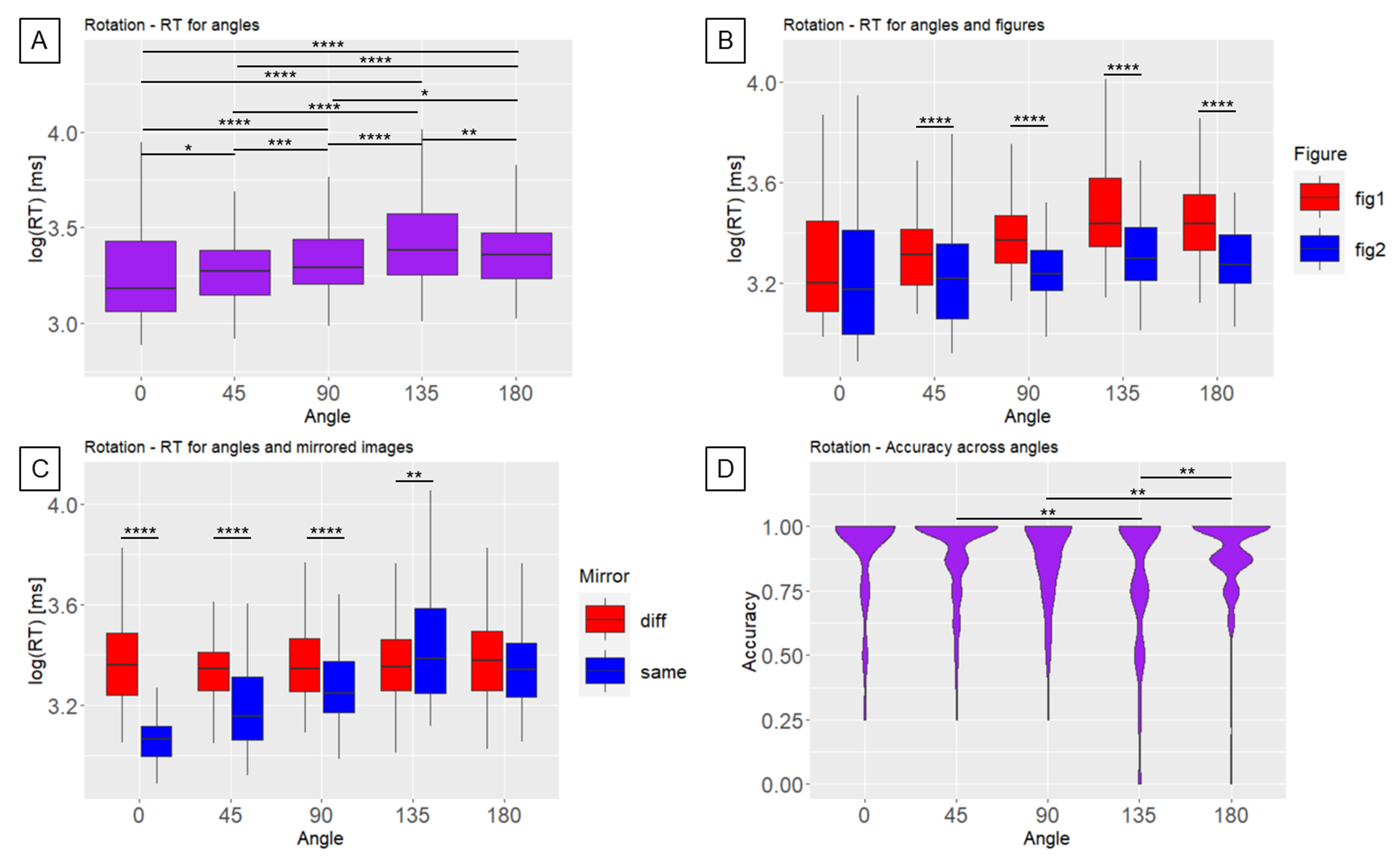

Supplement: S3 Fig — Mental Rotation task reaction time (RT) as a function of (A) rotation angle, (B) rotation angle and figure type (fig1 and fig2 = unfamiliar and familiar figures, respectively), and (C) on rotation angle and mirroring (diff and same represent mirrored and unmirrored figures, respectively). (D) Mental Rotation task accuracy (number of correct responses divided by total number of trials) as a function of rotation angle. Asterisks represent significant differences in post-hoc tests corrected for multiple comparisons using the Sidak method (**** p < 0.0001, *** p < 0.001, ** p < 0.01, * p < 0.05). (TIF) [file pone.0281196.s003.tif]

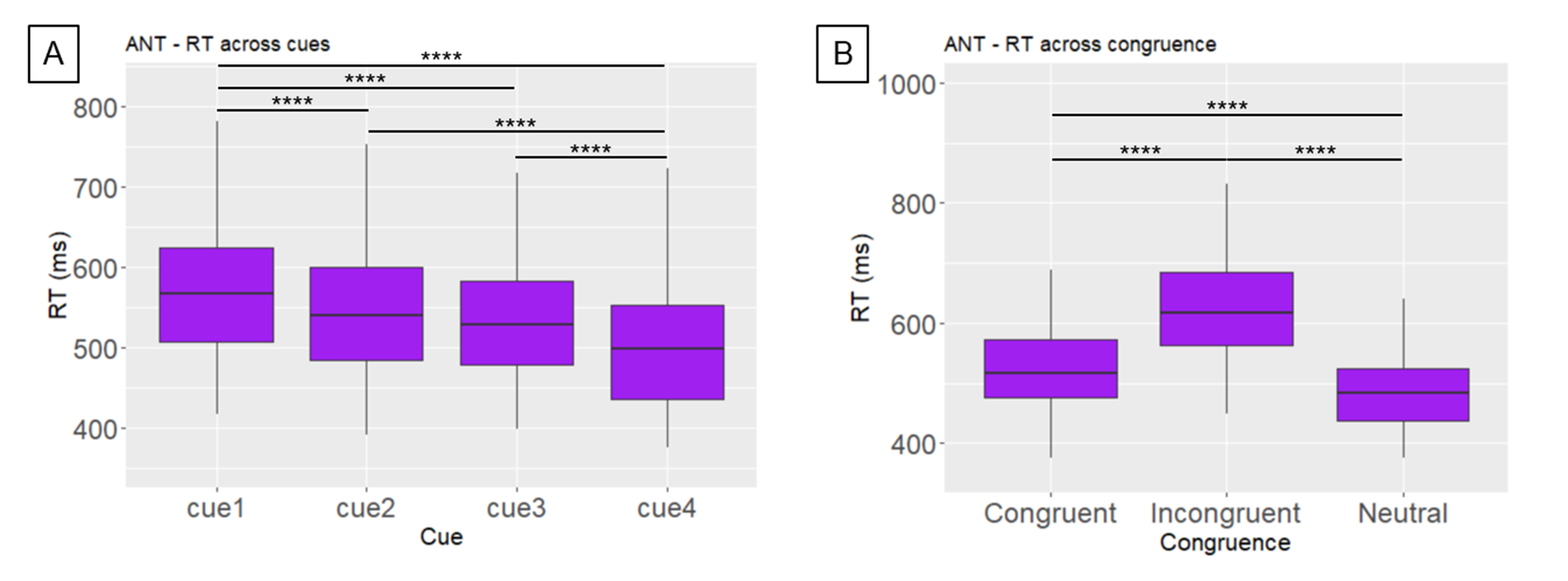

Supplement: S4 Fig — ANT reaction time (RT) scores as a function of (A) cue type (cue1, cue2, cue3, and cue4 represent uncued, center cued, top-bottom cued, and direction-cued trials, respectively) and (B) stimulus congruence. Asterisks represent significant differences in post-hoc tests corrected for multiple comparisons using the Sidak method (**** p < 0.0001). (TIF) [file pone.0281196.s004.tif]

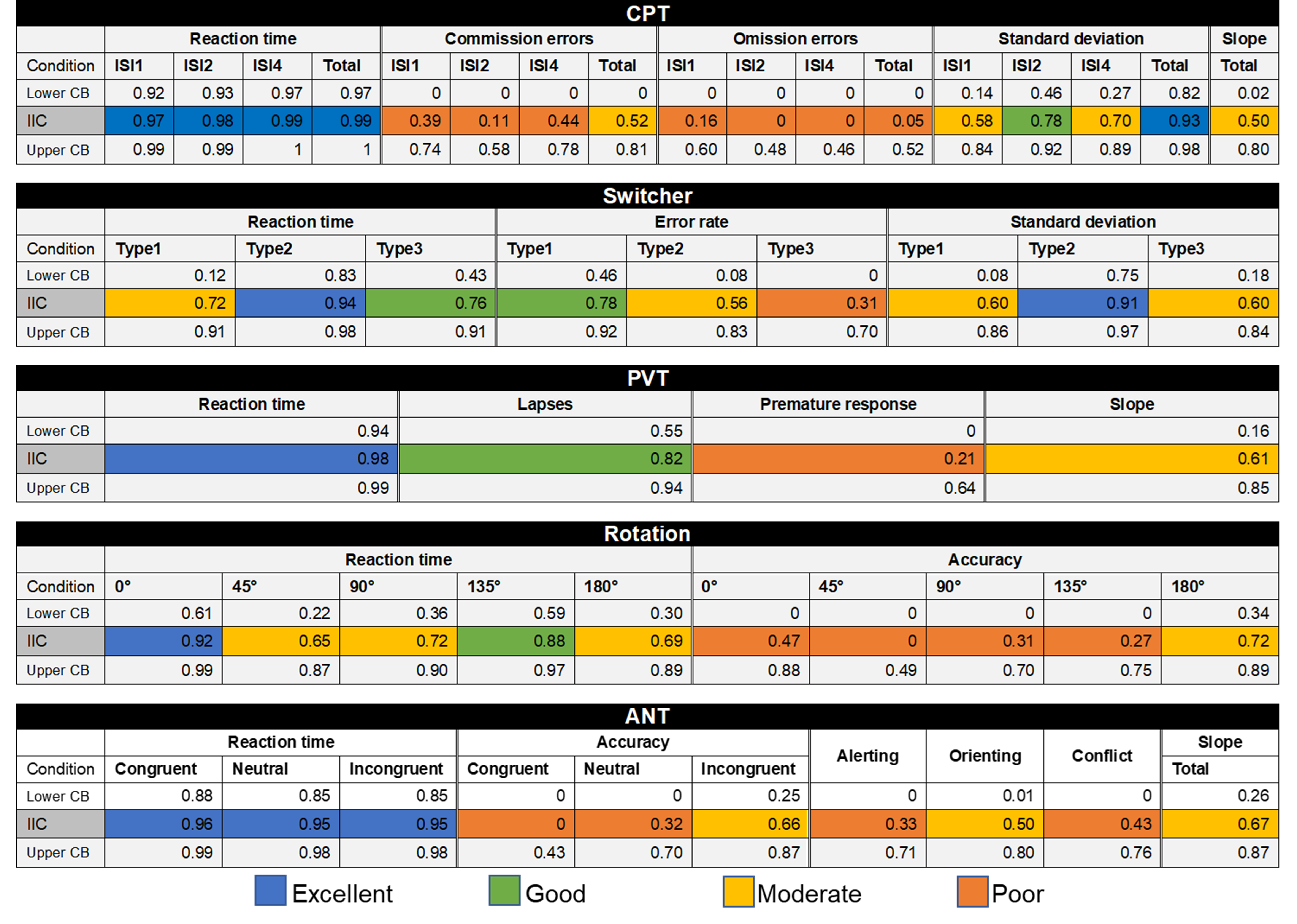

Supplement: S5 Fig — CPT = Continuous Performance Task; PVT = Psychomotor Vigilance Task; ANT = Attention Network Test; ISI1, ISI2, ISI4 = inter-stimulus interval of 1, 2, and 4 s, respectively; Type1, Type2, and Type3 = alternate switch, fixed switch, and random switch, respectively, CB = confidence boundary. Blue, green, yellow, and orange colors indicate excellent (ICC ≥ 0.9), good (0.75 ≤ ICC < 0.9), moderate (0.5 ≤ ICC < 0.75), and poor (ICC < 0.5) ICC values. (TIF) [file pone.0281196.s005.tif]

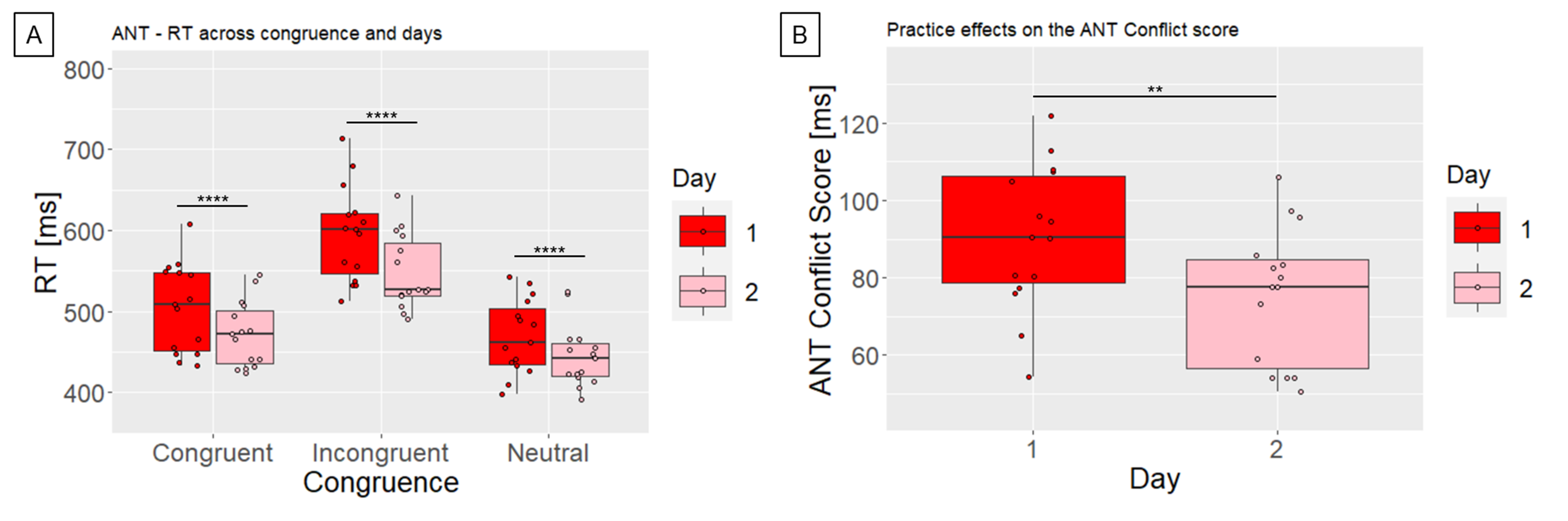

Supplement: S6 Fig — (A) ANT RT scores as a function of stimulus congruence and measurement session (day). (B) Attentional Network Test (ANT) conflict score as a function of measurement session. Day 1 = first application, Day 2 = repeated application (i.e., after practice). Asterisks represent significant differences in post-hoc tests corrected for multiple comparisons using the Sidak method (**** p < 0.0001, ** p < 0.01). (TIF) [file pone.0281196.s006.tif]

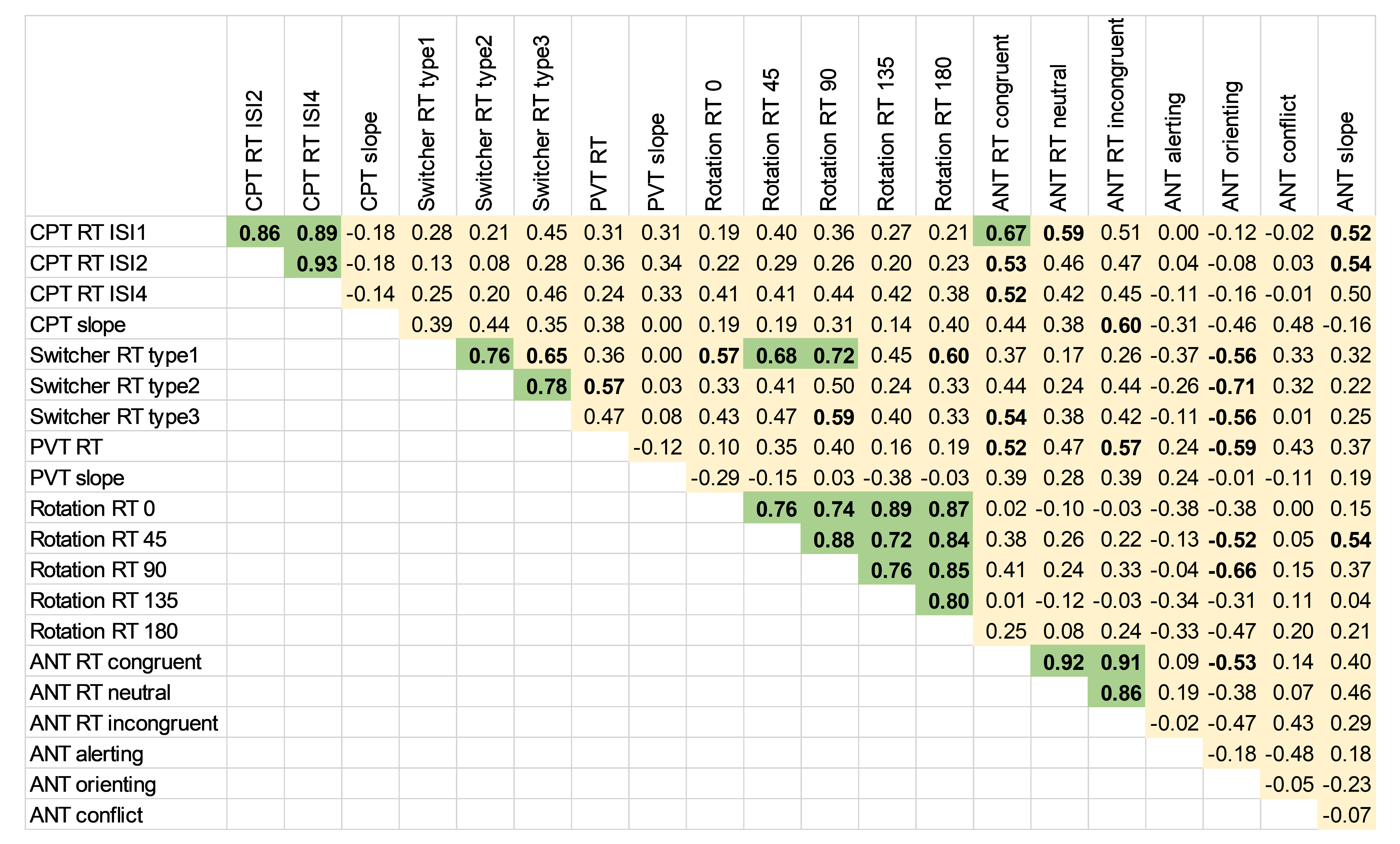

Supplement: S7 Fig — Significant positive and negative correlations (FDR-corr. p < 0.05) are reported in green and red, respectively. Correlations with p < 0.05, uncorrected for multiple comparisons, are printed in bold. CPT = Continuous Performance Task; PVT = Psychomotor Vigilance Task; ANT = Attention Network Test; RT = reaction time; ISI1, ISI2, ISI4 = inter-stimulus interval of 1, 2, and 4 s, respectively; type1, type2, type3 = alternate switch, fixed switch, random switch, respectively. (TIF) [file pone.0281196.s007.tif]

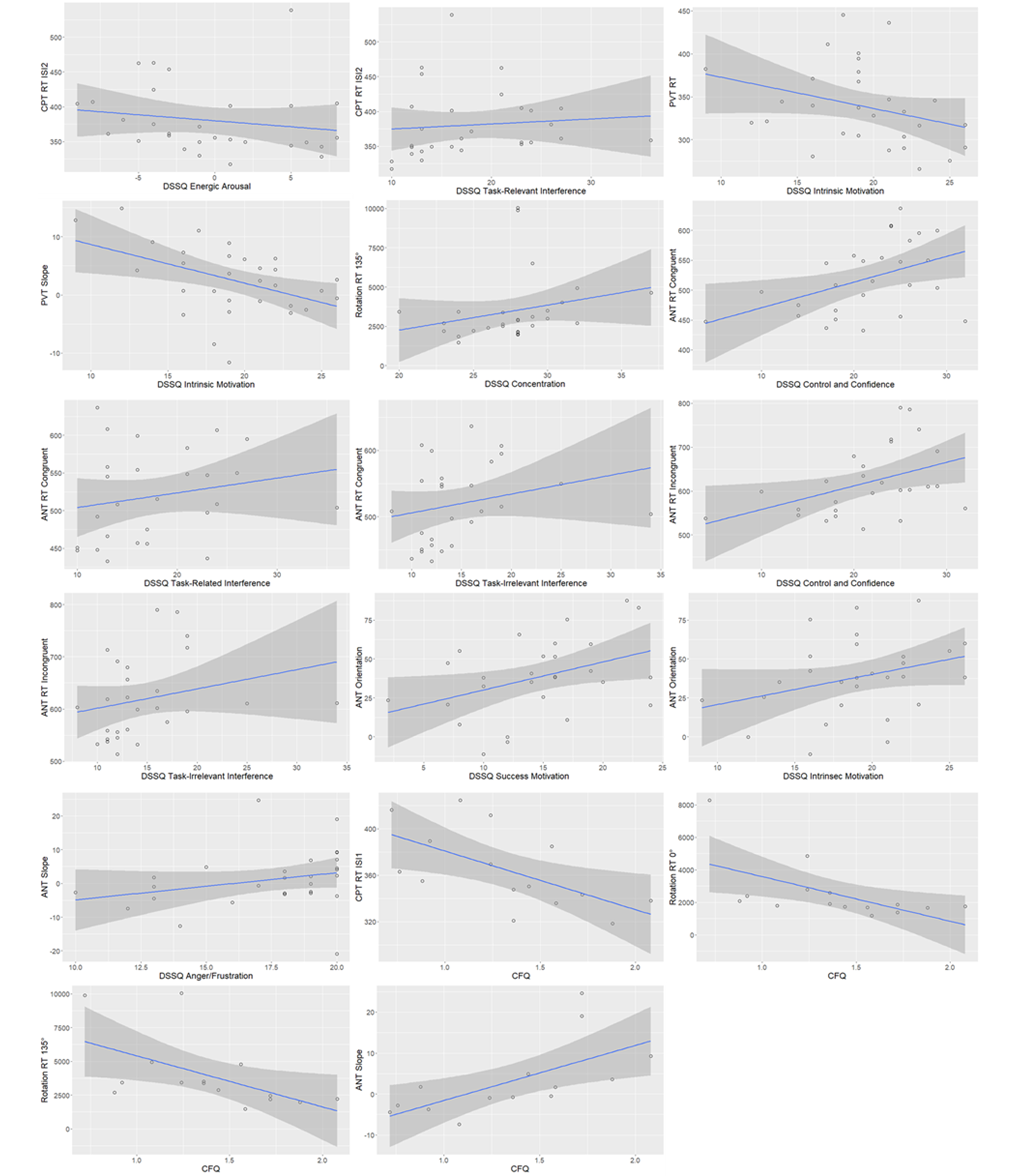

Supplement: S8 Fig — CPT = Continuous Performance Task; RT = reaction time; ISI1 and ISI2 = inter-stimulus interval of 1 and 2 s, respectively; DSSQ = Dundee Stress State Questionnaire; PVT = Psychometric Vigilance Task; ANT = Attentional Network Test; CFQ = Cognitive Failures Questionnaire. (TIF) [file pone.0281196.s008.tif]
