# Supplementary material for: Evaluation of the reliability and validity of computerized tests of attention
Source: PLoS One. 2023 Jan 27;18(1):e0281196. doi: 10.1371/journal.pone.0281196 (PMC9882756; doi:10.1371/journal.pone.0281196)
Supplement: S1 Table — (DOCX) [file pone.0281196.s009.docx]

**S1 Table.**

Statistics of significant main effects and interactions across conditions within tasks and post-hoc analyses

| ***CPT***^a^ | | | | | | | | | | | |
| --- | --- | --- | --- | --- | --- | --- | --- | --- | --- | --- | --- |
| **Reaction time – Main effect ISI – F(2,98) = 122.4, η² = 0.71, p < 0.0001** | | | | | | | | | | | |
| ISI^b^ | | Difference (ms) | | | DoF^c^ | t-value | | Cohen’s d | | p-value ^d^ | |
| ISI1 - ISI2 | | -24.0 | | | 98 | -7.24 | | 1.08 | | <0.0001 | |
| ISI1 - ISI4 | | -53.3 | | |  | -15.6 | | 1.76 | | <0.0001 | |
| ISI2 - ISI4 | | -29.3 | | |  | -8.36 | | 1.59 | | <0.0001 | |
| **Standard deviation – Main effect ISI – F(2,100) = 3.49, η² = 0.07, p = 0.03** | | | | | | | | | | | |
| ISI | | Difference (ms) | | | DoF | t-value | | Cohen’s d | | p-value | |
| ISI1 – ISI2 | | 7.75 | | | 100 | 2.74 | | 0.41 | | 0.020 | |
| ***Switcher*** | | | | | | | | | | | |
| **Standard deviation – Main effect Type – F(2,96) = 5.53, η² = 0.10, p = 0.005** | | | | | | | | | | | |
| Type^e^ | | Difference (ms) | | | DoF | t-value | | Cohen’s d | | p-value | |
| Type1 – Type2 | | -98.1 | | | 96 | -3.54 | | 0.46 | | 0.0018 | |
| Type1 – Type3 | | -75.9 | | |  | -2.74 | | 0.29 | | 0.02 | |
| ***Rotation*** | | | | | | | | | | | |
| **Reaction time – Interaction Mirror x Figure x Angle – F(4,120) = 5.93, η² = 0.17, p = 0.0002** | | | | | | | | | | | |
| No significant post-hoc differences. | | | | | | | | | | | |
| **Reaction time – Interaction Figure x Angle – F(4,120) = 5.30, η² = 0.15, p = 0.0006** | | | | | | | | | | | |
| Angle | Figure | | Difference (ms) | DoF | | | t-value | | Cohen’s d | | p-value |
| 45° | Fig1 - Fig2^f^ | | 368 | 117 | | | 4.58 | | 0.80 | | <0.0001 |
| 90° |  |  | 606 |  |  |  | 4.80 | | 0.81 | | <0.0001 |
| 135° |  |  | 924 |  |  |  | 6.56 | | 0.64 | | <0.0001 |
| 180° |  |  | 889 |  |  |  | 5.57 | | 1.08 | | <0.0001 |
| 0° - 45° | Fig1 | | -368 | 238 | | | -3.89 | | 0.53 | | 0.0012 |
| 0° - 90° |  |  | -692 |  |  |  | -6.98 | | 0.82 | | <0.0001 |
| 0° - 135° |  |  | -1342 |  |  |  | -12.39 | | 1.07 | | <0.0001 |
| 0° - 180° |  |  | -1064 |  |  |  | -10.0 | | 1.06 | | <0.0001 |
| 45° - 90° |  |  | -324 |  |  |  | -3.09 | | 0.51 | | 0.02 |
| 45° - 135° |  |  | -975 |  |  |  | -8.50 | | 0.78 | | <0.0001 |
| 45° - 180° |  |  | -889 |  |  |  | -6.13 | | 1.08 | | <0.0001 |
| 90° - 135° |  |  | -650 |  |  |  | -5.40 | | 0.59 | | <0.0001 |
| 90° - 180° |  |  | -372 |  |  |  | -3.03 | | 0.45 | | 0.022 |
| 0° - 90° | Fig2 | | -246 | 238 | | | -3.33 | | 0.41 | | 0.009 |
| 0° - 135° |  |  | -579 |  |  |  | -6.59 | | 0.50 | | <0.0001 |
| 0° - 180° |  |  | -336 |  |  |  | -4.12 | | 0.39 | | 0.0005 |
| 45° - 90° |  |  | -207 |  |  |  | -2.83 | | 0.45 | | 0.04 |
| 45° - 135° |  |  | -540 |  |  |  | -6.09 | | 0.58 | | <0.0001 |
| 45° - 180° |  |  | -297 |  |  |  | -6.65 | | 0.44 | | 0.003 |
| 90° - 135° |  |  | -333 |  |  |  | -3.26 | | 0.34 | | 0.011 |
| **Reaction time – Interaction Mirror x Angle – F(4,120) = 63.7, η² = 0.68, p < 0.0001** | | | | | | | | | | | |
| Angle | Mirror | | Difference (ms) | DoF | | | t-value | | Cohen’s d | | p-value |
| 0° | diff - same^g^ | | 1139 | 130 | | | 15.7 | | 1.81 | | <0.0001 |
| 45° |  |  | 706 |  |  |  | 8.80 | | 1.17 | | <0.0001 |
| 90° |  |  | 505 |  |  |  | 5.14 | | 0.76 | | <0.0001 |
| 135° |  |  | -306 |  |  |  | -2.79 | | 0.26 | | 0.006 |
| 0° - 45° | same | | -326 | 236 | | | -6.04 | | 0.97 | | <0.0001 |
| 0° - 90° |  |  | -691 |  |  |  | -11.2 | | 1.57 | | <0.0001 |
| 0° - 135° |  |  | -1559 |  |  |  | -20.2 | | 1.96 | | <0.0001 |
| 0° - 180° |  |  | -1085 |  |  |  | -15.6 | | 1.86 | | <0.0001 |
| 45° - 90° |  |  | -365 |  |  |  | -5.20 | | 0.73 | | <0.0001 |
| 45° - 135° |  |  | -1233 |  |  |  | -14.1 | | 1.39 | | <0.0001 |
| 45° - 180° |  |  | -759 |  |  |  | -9.51 | | 1.07 | | <0.0001 |
| 90° - 135° |  |  | -869 |  |  |  | -8.93 | | 1.04 | | <0.0001 |
| 90° - 180° |  |  | -394 |  |  |  | -4.31 | | 0.63 | | 0.0002 |
| 135° - 180° |  |  | 474 |  |  |  | 4.62 | | 0.55 | | 0.0001 |
| **Reaction time – Main effect Angle – F(4,120) = 62.5, η² = 0.68, p < 0.0001** | | | | | | | | | | | |
| Angle | | | Difference (ms) | DoF | | | t-value | | Cohen’s d | | p-value |
| 0° - 45° | | | -168 | 120 | | | -3.24 | | 0.32 | | 0.013 |
| 0° - 90° | | | -450 |  |  |  | -7.63 | | 0.61 | | <0.0001 |
| 0° - 135° | | | -954 |  |  |  | -14.0 | | 0.75 | | <0.0001 |
| 0° - 180° | | | -661 |  |  |  | -10.5 | | 0.68 | | <0.0001 |
| 45° - 90° | | | -282 |  |  |  | -4.38 | | 0.48 | | 0.0002 |
| 45° - 135° | | | -786 |  |  |  | -10.8 | | 0.68 | | <0.0001 |
| 45° - 180° | | | -493 |  |  |  | -7.22 | | 0.59 | | <0.0001 |
| 90° - 135° | | | -504 |  |  |  | -6.41 | | 0.46 | | <0.0001 |
| 90° - 180° | | | -211 |  |  |  | -2.83 | | 0.29 | | 0.04 |
| 135° - 180° | | | 293 |  |  |  | 3.58 | | 0.30 | | 0.004 |
| **Reaction time – Main effect Figure – F(1,30) = 57.8, η² = 0.66, p < 0.0001** | | | | | | | | | | | |
| Figure | | | Difference (ms) | DoF | | | t-value | | Cohen’s d | | p-value |
| Fig1 - Fig2 | | | 549 | 30 | | | 7.60 | | 0.66 | | <0.0001 |
| **Reaction time – Main effect Mirror – F(1,11) = 87.1, η² = 0.74, p < 0.0001** | | | | | | | | | | | |
| Mirror | | | Difference (ms) | DoF | | | t-value | | Cohen’s d | | p-value |
| diff - same | | | 493 | 30 | | | 9.33 | | 0.54 | | <0.0001 |
| **Accuracy – Main effect Angle – X²(4) = 26.8, p < 0.0001** | | | | | | | | | | | |
| Angle | | | | Difference (%) | | | DoF | | z-value | | p-value |
| 45° - 135° | | | | 1.67 | | | Inf | | 3.50 | | 0.004 |
| 90° - 135° | | | | 1.67 | | |  |  | 3.50 | | 0.004 |
| 135° - 180° | | | | -1.86 | | |  |  | -3.65 | | 0.0025 |
| ***ANT*** | | | | | | | | | | | |
| **Reaction time – Interaction Cue x Congruence – F(6,174) = 9.47, η² = 0.25, p < 0.0001** | | | | | | | | | | | |
| Cue | Congruence | | Difference (ms) | DoF | | | t-value | | Cohen’s d | | p-value |
| cue1 - cue2^h^ | Congruent | | 38.2 | 204 | | | 6.65 | | 1.44 | | <0.0001 |
| cue1 - cue3 |  |  | 35.4 |  |  |  | 6.18 | | 1.29 | | <0.0001 |
| cue1 - cue4 |  |  | 64.8 |  |  |  | 11.3 | | 2.07 | | <0.0001 |
| cue2 - cue4 |  |  | 26.6 |  |  |  | 4.64 | | 1.04 | | <0.0001 |
| cue3 - cue4 |  |  | 29.4 |  |  |  | 5.12 | | 1.07 | | <0.0001 |
| cue1 - cue2 | Neutral | | 42.1 | 204 | | | 7.34 | | 1.37 | | <0.0001 |
| cue1 - cue3 |  |  | 46.5 |  |  |  | 8.12 | | 1.34 | | <0.0001 |
| cue1 - cue4 |  |  | 70.1 |  |  |  | 12.2 | | 1.75 | | <0.0001 |
| cue2 - cue4 |  |  | 28.0 |  |  |  | 4.88 | | 0.95 | | <0.0001 |
| cue3 - cue4 |  |  | 23.6 |  |  |  | 4.11 | | 1.05 | | 0.0003 |
| cue1 - cue3 | Incongruent | | 18.8 | 204 | | | 3.27 | | 0.55 | | 0.007 |
| cue1 - cue4 |  |  | 71.4 |  |  |  | 12.5 | | 1.72 | | <0.0001 |
| cue2 - cue4 |  |  | 61.3 |  |  |  | 10.7 | | 1.52 | | <0.0001 |
| cue3 - cue4 |  |  | 52.7 |  |  |  | 9.18 | | 1.28 | | <0.0001 |
| Cue1 | con – inc^i^ | | -92.1 | 112 | | | -12.9 | | 2.46 | | <0.0001 |
|  | con – neu | | 35.0 |  |  |  | 4.90 | | 1.01 | | <0.0001 |
|  | inc – neu | | 127 |  |  |  | 17.8 | | 2.43 | | <0.0001 |
| Cue2 | con - inc | | -120 | 112 | | | -16.8 | | 3.56 | | <0.0001 |
|  | con - neu | | 38.9 |  |  |  | 5.46 | | 1.70 | | <0.0001 |
|  | inc – neu | | 159 |  |  |  | 22.3 | | 3.47 | | <0.0001 |
| Cue3 | con - inc | | -109 | 112 | | | -15.32 | | 2.49 | | <0.0001 |
|  | con - neu | | 46.1 |  |  |  | 6.46 | | 2.12 | | <0.0001 |
|  | inc – neu | | 155 |  |  |  | 21.7 | | 3.17 | | <0.0001 |
| Cue4 | con - inc | | -85.4 | 112 | | | -12.0 | | 2.20 | | <0.0001 |
|  | con - neu | | 40.3 |  |  |  | 5.65 | | 1.52 | | <0.0001 |
|  | inc – neu | | 126 |  |  |  | 17.6 | | 2.65 | | <0.0001 |
| **Reaction time – Main effect Congruence – F(2,58) = 300.6, η² = 0.91, p < 0.0001** | | | | | | | | | | | |
| Congruence | | | Difference (ms) | DoF | | | t-value | | Cohen’s d | | p-value |
| con – neu | | | 40.1 | 58 | | | 6.73 | | 1.49 | | <0.0001 |
| con – inc | | | -102 |  |  |  | -17.1 | | 2.51 | | <0.0001 |
| neu – inc | | | -141.7 |  |  |  | -23.8 | | 2.81 | | <0.0001 |
| **Reaction time – Main effect Cue – F(2,58) = 300.6, η² = 0.74, p < 0.0001** | | | | | | | | | | | |
| Cue | | | Difference (ms) | DoF | | | t-value | | Cohen’s d | | p-value |
| cue1 - cue2 | | | 30.1 | 87 | | | 6.88 | | 0.89 | | <0.0001 |
| cue1 - cue3 | | | 33.6 |  |  |  | 7.66 | | 0.99 | | <0.0001 |
| cue1 - cue4 | | | 68.8 |  |  |  | 15.7 | | 1.83 | | <0.0001 |
| cue2 - cue4 | | | 38.6 |  |  |  | 8.82 | | 1.08 | | <0.0001 |
| cue3 - cue4 | | | 35.2 |  |  |  | 8.04 | | 1.05 | | <0.0001 |

*Note. ^a^CPT = Continuous Performance Task; ^b^ISI1, ISI2, ISI4 = inter-stimulus intervals for 1, 2, 4 s, respectively; ^c^DoF = degrees-of-freedom; ^d^P-values adjusted (for multiple comparisons) using the Sidak method; ^e^Type1, Type2, Type3 = alternate switch, fixed switch, random switch, respectively; ^f^Fig1, Fig2 = unfamiliar and familiar figures, respectively; ^g^diff, same = mirrored and unmirrored figures, respectively; ^h^cue1, cue2, cue3, cue4 = uncued, center cued, top-bottom cued, and direction cued stimuli, respectively; ^i^coh, neu, inc = congruent, neutral, incongruent, respectively.*
