# Supplementary material for: Evaluation of the reliability and validity of computerized tests of attention
Source: PLoS One. 2023 Jan 27;18(1):e0281196. doi: 10.1371/journal.pone.0281196 (PMC9882756; doi:10.1371/journal.pone.0281196)
Supplement: S2 Table — (DOCX) [file pone.0281196.s010.docx]

**S2 Table.**

Statistics of significant main effects and interactions and post-hoc analyses for double application (Study II)

| ***Switcher – only second day*** | | | | | |  |
| --- | --- | --- | --- | --- | --- | --- |
| **Reaction time – Main effect Type – F(2,26) = 4.12, η² = 0.24, p = 0.028** | | | | | |  |
| Type^a^ | Difference (ms) | DoF^b^ | t-value | Cohen’s d | p-value | |
| Type1 - Type3 | -142.7 | 26 | -2.66 | 0.66 | 0.03 | |

Note. P-values are adjusted for multiple comparisons using the Sidak method. ^a^Type1, Type2, Type3 = alternate switch, fixed switch, random switch, respectively; ^b^DoF = degrees-of-freedom.
