# Supplementary material for: Evaluation of the reliability and validity of computerized tests of attention
Source: PLoS One. 2023 Jan 27;18(1):e0281196. doi: 10.1371/journal.pone.0281196 (PMC9882756; doi:10.1371/journal.pone.0281196)
Supplement: S3 Table — (DOCX) [file pone.0281196.s011.docx]

**S3 Table.**

Descriptive statistics for performance measures of the CPT (Continuous Performance Task)

| **Score** | **Condition** | **Study** | **Day** | **N** | **Mean** | **SD** | **Min** | **Max** |
| --- | --- | --- | --- | --- | --- | --- | --- | --- |
| Reaction Time (ms) | ISI1 | 1 | 1 | 16 | 365.83 | 33.15 | 318.38 | 424.44 |
|  |  | 2 | 1 | 14 | 348.07 | 38.56 | 302.56 | 440.57 |
|  |  |  | 2 | 14 | 320.67 | 39.79 | 279.32 | 408.20 |
|  |  | 3 | 1 | 19 | 336.76 | 32.43 | 276.69 | 392.12 |
|  | ISI2 | 1 | 1 | 16 | 383.27 | 37.73 | 317.19 | 453.55 |
|  |  | 2 | 1 | 14 | 374.51 | 48.16 | 327.97 | 462.94 |
|  |  |  | 2 | 14 | 347.73 | 48.81 | 282.41 | 459.44 |
|  |  | 3 | 1 | 19 | 359.08 | 34.66 | 287.76 | 418.97 |
|  | ISI4 | 1 | 1 | 16 | 417.91 | 54.37 | 357.51 | 542.96 |
|  |  | 2 | 1 | 14 | 393.20 | 37.43 | 362.45 | 463.94 |
|  |  |  | 2 | 14 | 378.20 | 39.39 | 308.62 | 457.12 |
|  |  | 3 | 1 | 19 | 391.06 | 43.28 | 290.63 | 457.79 |
|  | Total | 1 | 1 | 16 | 389.06 | 37.58 | 331.02 | 460.45 |
|  |  | 2 | 1 | 14 | 371.93 | 39.47 | 333.65 | 449.56 |
|  |  |  | 2 | 14 | 348.87 | 41.57 | 290.12 | 441.59 |
|  |  | 3 | 1 | 19 | 362.30 | 34.65 | 285.02 | 409.44 |
| Commission Errors (%) | ISI1 | 1 | 1 | 16 | 37.50 | 19.00 | 8.33 | 75.00 |
|  |  | 2 | 1 | 15 | 30.00 | 14.93 | 8.33 | 63.64 |
|  |  |  | 2 | 15 | 37.58 | 17.16 | 8.33 | 63.64 |
|  |  | 3 | 1 | 19 | 28.63 | 17.62 | 0.00 | 58.33 |
|  | ISI2 | 1 | 1 | 16 | 31.77 | 16.16 | 8.33 | 58.33 |
|  |  | 2 | 1 | 15 | 33.89 | 15.58 | 8.33 | 58.33 |
|  |  |  | 2 | 15 | 39.14 | 25.86 | 8.33 | 81.82 |
|  |  | 3 | 1 | 19 | 36.08 | 18.81 | 0 | 66.67 |
|  | ISI4 | 1 | 1 | 16 | 40.34 | 20.73 | 0 | 75.00 |
|  |  | 2 | 1 | 15 | 38.89 | 14.42 | 8.33 | 58.33 |
|  |  |  | 2 | 15 | 36.67 | 20.12 | 8.33 | 91.67 |
|  |  | 3 | 1 | 19 | 34.49 | 20.40 | 0.00 | 75.00 |
|  | Total | 1 | 1 | 16 | 36.55 | 14.32 | 13.89 | 58.33 |
|  |  | 2 | 1 | 15 | 34.21 | 11.38 | 8.33 | 51.43 |
|  |  |  | 2 | 15 | 37.73 | 18.13 | 11.11 | 77.14 |
|  |  | 3 | 1 | 19 | 33.05 | 16.19 | 5.56 | 61.11 |
| Omission Errors (%) | ISI1 | 1 | 1 | 16 | 0.89 | 1.07 | 0 | 2.94 |
|  |  | 2 | 1 | 15 | 0.69 | 1.09 | 0 | 3.74 |
|  |  |  | 2 | 15 | 0.69 | 0.75 | 0 | 1.89 |
|  |  | 3 | 1 | 19 | 0.64 | 0.98 | 0 | 3.70 |
|  | ISI2 | 1 | 1 | 16 | 0.48 | 1.68 | 0 | 6.73 |
|  |  | 2 | 1 | 15 | 0.19 | 0.52 | 0 | 1.85 |
|  |  |  | 2 | 15 | 0.45 | 0.63 | 0 | 1.98 |
|  |  | 3 | 1 | 19 | 0.40 | 0.66 | 0 | 1.92 |
|  | ISI4 | 1 | 1 | 16 | 0.53 | 1.02 | 0 | 2.83 |
|  |  | 2 | 1 | 15 | 0.13 | 0.33 | 0 | 0.95 |
|  |  |  | 2 | 15 | 0.19 | 0.39 | 0 | 0.95 |
|  |  | 3 | 1 | 19 | 0.25 | 0.54 | 0 | 1.92 |
|  | Total | 1 | 1 | 16 | 0.63 | 0.77 | 0 | 3.21 |
|  |  | 2 | 1 | 15 | 0.34 | 0.50 | 0 | 1.58 |
|  |  |  | 2 | 15 | 0.44 | 0.35 | 0 | 0.95 |
|  |  | 3 | 1 | 19 | 0.43 | 0.49 | 0 | 1.58 |
| Slope | Total | 1 | 1 | 16 | 2.72 | 8.30 | -7.24 | 25.36 |
|  |  | 2 | 1 | 15 | 2.62 | 7.73 | -4.87 | 20.60 |
|  |  |  | 2 | 15 | -1.70 | 5.14 | -13.25 | 4.54 |
|  |  | 3 | 1 | 19 | 1.10 | 4.50 | -4.82 | 10.05 |

*Note. N = sample size, SD = standard deviation, Min = minimum, Max = maximum; ISI1, ISI2, ISI4 = inter-stimulus interval of 1, 2, and 4 s, respectively.*
