# Supplementary material for: Evaluation of the reliability and validity of computerized tests of attention
Source: PLoS One. 2023 Jan 27;18(1):e0281196. doi: 10.1371/journal.pone.0281196 (PMC9882756; doi:10.1371/journal.pone.0281196)
Supplement: S4 Table — (DOCX) [file pone.0281196.s012.docx]

**S4 Table.**

Descriptive statistics for performance measures of the Switcher Task

| **Score** | **Condition** | **Study** | **Day** | **N** | **Mean** | **SD** | **Min** | **Max** |
| --- | --- | --- | --- | --- | --- | --- | --- | --- |
| Reaction Time (ms) | Type1 | 1 | 1 | 16 | 1999.65 | 282.91 | 1614.23 | 2587.44 |
|  |  | 2 | 1 | 14 | 1930.47 | 337.28 | 1525.44 | 2820.57 |
|  |  |  | 2 | 14 | 1625.69 | 302.78 | 1254.19 | 2323.85 |
|  |  | 3 | 1 | 20 | 2002.08 | 246.70 | 1437.91 | 2687.53 |
|  | Type2 | 1 | 1 | 16 | 1920.35 | 223.78 | 1625.14 | 2299.78 |
|  |  | 2 | 1 | 14 | 1943.79 | 481.07 | 1352.09 | 3413.13 |
|  |  |  | 2 | 14 | 1649.15 | 295.34 | 1302.89 | 2388.88 |
|  |  | 3 | 1 | 20 | 2022.19 | 262.23 | 1466.63 | 2580.74 |
|  | Type3 | 1 | 1 | 16 | 1924.77 | 201.87 | 1609.73 | 2264.92 |
|  |  | 2 | 1 | 14 | 1955.26 | 352.69 | 1510.00 | 2918.32 |
|  |  |  | 2 | 14 | 1731.73 | 230.39 | 1332.19 | 2274.85 |
|  |  | 3 | 1 | 20 | 2079.33 | 291.78 | 1699.40 | 2858.62 |
| Errors (%) | Type1 | 1 | 1 | 16 | 1.70 | 2.55 | 0 | 7.69 |
|  |  | 2 | 1 | 15 | 1.43 | 2.66 | 0 | 8.11 |
|  |  |  | 2 | 15 | 2.29 | 3.99 | 0 | 10.81 |
|  |  | 3 | 1 | 20 | 2.31 | 3.02 | 0 | 10.53 |
|  | Type2 | 1 | 1 | 16 | 2.79 | 4.22 | 0 | 15.00 |
|  |  | 2 | 1 | 15 | 2.27 | 3.55 | 0 | 12.50 |
|  |  |  | 2 | 15 | 2.42 | 4.16 | 0 | 14.63 |
|  |  | 3 | 1 | 20 | 1.86 | 3.21 | 0 | 10.53 |
|  | Type3 | 1 | 1 | 16 | 2.64 | 3.60 | 0 | 12.50 |
|  |  | 2 | 1 | 15 | 1.83 | 2.44 | 0 | 7.89 |
|  |  |  | 2 | 15 | 2.49 | 3.90 | 0 | 10.53 |
|  |  | 3 | 1 | 20 | 2.27 | 2.83 | 0 | 10.26 |

*Note. N = sample size, SD = standard deviation, Min = minimum, Max = maximum; Type1, Type2, Type3 = alternate switch, fixed switch, random switch, respectively.*
