# Supplementary material for: Evaluation of the reliability and validity of computerized tests of attention
Source: PLoS One. 2023 Jan 27;18(1):e0281196. doi: 10.1371/journal.pone.0281196 (PMC9882756; doi:10.1371/journal.pone.0281196)
Supplement: S5 Table — (DOCX) [file pone.0281196.s013.docx]

**S5 Table.**

Descriptive statistics for performance measures of the PVT (Psychomotor Vigilance Task)

| **Score** | **Study** | **Day** | **N** | **Mean** | **SD** | **Min** | **Max** |
| --- | --- | --- | --- | --- | --- | --- | --- |
| Reaction Time (ms) | 1 | 1 | 17 | 352.45 | 53.44 | 275.75 | 480.59 |
|  | 2 | 1 | 15 | 331.35 | 50.51 | 265.76 | 445.25 |
|  |  | 2 | 15 | 327.73 | 53.86 | 263.50 | 444.15 |
|  | 3 | 1 | 20 | 421.21 | 56.33 | 317.35 | 521.07 |
| Lapses | 1 | 1 | 17 | 5.65 | 11.61 | 0 | 47 |
|  | 2 | 1 | 15 | 2.40 | 5.57 | 0 | 20 |
|  |  | 2 | 15 | 2.53 | 5.11 | 0 | 17 |
|  | 3 | 1 | 20 | 20.60 | 19.39 | 0 | 67 |
| Premature responses | 1 | 1 | 17 | 3.24 | 5.37 | 0 | 21 |
|  | 2 | 1 | 15 | 1.53 | 1.85 | 0 | 7 |
|  |  | 2 | 15 | 1.33 | 1.54 | 0 | 5 |
|  | 3 | 1 | 20 | 0.60 | 0.75 | 0 | 2 |
| Slope | 1 | 1 | 17 | 3.16 | 6.30 | -11.65 | 17.46 |
|  | 2 | 1 | 15 | 2.76 | 6.55 | -8.46 | 14.87 |
|  |  | 2 | 15 | 0.05 | 6.65 | -17.89 | 12.62 |
|  | 3 | 1 | 20 | 0.42 | 9.88 | -15.30 | 23.88 |

*Note. N = sample size, SD = standard deviation, Min = minimum, Max = maximum.*
