# Supplementary material for: Evaluation of the reliability and validity of computerized tests of attention
Source: PLoS One. 2023 Jan 27;18(1):e0281196. doi: 10.1371/journal.pone.0281196 (PMC9882756; doi:10.1371/journal.pone.0281196)
Supplement: S6 Table — (DOCX) [file pone.0281196.s014.docx]

**S6 Table.**

Descriptive statistics for performance measures of the Mental Rotation task

| **Score** | **Condition** | **Study** | **Day** | **N** | **Mean** | **SD** | **Min** | **Max** |
| --- | --- | --- | --- | --- | --- | --- | --- | --- |
| Reaction Time (ms) | 0° | 1 | 1 | 17 | 2282.38 | 1733.62 | 778.50 | 8843.50 |
|  |  | 2 | 1 | 15 | 1785.17 | 864.34 | 772.00 | 3872.50 |
|  |  |  | 2 | 15 | 1496.45 | 657.68 | 666.00 | 4055.50 |
|  | 45° | 1 | 1 | 17 | 2434.79 | 1797.51 | 848.00 | 8780.50 |
|  |  | 2 | 1 | 15 | 1927.77 | 744.33 | 831.50 | 4860.00 |
|  |  |  | 2 | 15 | 1631.75 | 677.24 | 794.50 | 4005.50 |
|  | 90° | 1 | 1 | 17 | 2589.43 | 1798.63 | 971.50 | 10060.00 |
|  |  | 2 | 1 | 15 | 2313.31 | 979.20 | 1076.00 | 5782.00 |
|  |  |  | 2 | 15 | 1937.44 | 909.62 | 965.00 | 6017.00 |
|  | 135° | 1 | 1 | 17 | 3523.40 | 2598.98 | 1144.00 | 12776.50 |
|  |  | 2 | 1 | 15 | 2779.54 | 1704.19 | 1029.50 | 11397.00 |
|  |  |  | 2 | 15 | 2295.85 | 1134.92 | 1080.00 | 6725.50 |
|  | 180° | 1 | 1 | 17 | 2880.85 | 1847.39 | 1065.50 | 10736.00 |
|  |  | 2 | 1 | 15 | 2466.08 | 978.45 | 1125.00 | 5566.50 |
|  |  |  | 2 | 15 | 2161.28 | 1030.41 | 1075.50 | 6011.50 |
| Accuracy (%) | 0° | 1 | 1 | 17 | 89.84 | 19.78 | 25.00 | 100.00 |
|  |  | 2 | 1 | 15 | 92.92 | 17.28 | 25.00 | 100.00 |
|  |  |  | 2 | 15 | 96.67 | 10.76 | 50.00 | 100.00 |
|  | 45° | 1 | 1 | 17 | 85.76 | 19.22 | 25.00 | 100.00 |
|  |  | 2 | 1 | 15 | 94.99 | 9.09 | 62.50 | 100.00 |
|  |  |  | 2 | 15 | 93.21 | 10.05 | 62.50 | 100.00 |
|  | 90° | 1 | 1 | 17 | 87.34 | 16.02 | 42.86 | 100.00 |
|  |  | 2 | 1 | 15 | 88.41 | 15.68 | 25.00 | 100.00 |
|  |  |  | 2 | 15 | 91.10 | 12.71 | 50.00 | 100.00 |
|  | 135° | 1 | 1 | 17 | 80.99 | 25.61 | 0.00 | 100.00 |
|  |  | 2 | 1 | 15 | 80.83 | 26.59 | 0.00 | 100.00 |
|  |  |  | 2 | 15 | 83.89 | 26.48 | 0.00 | 100.00 |
|  | 180° | 1 | 1 | 17 | 87.79 | 14.55 | 28.57 | 100.00 |
|  |  | 2 | 1 | 15 | 89.51 | 19.70 | 0.00 | 100.00 |
|  |  |  | 2 | 15 | 86.01 | 18.88 | 25.00 | 100.00 |

*Note. N = sample size, SD = standard deviation, Min = minimum, Max = maximum.*
