# Supplementary material for: Evaluation of the reliability and validity of computerized tests of attention
Source: PLoS One. 2023 Jan 27;18(1):e0281196. doi: 10.1371/journal.pone.0281196 (PMC9882756; doi:10.1371/journal.pone.0281196)
Supplement: S7 Table — (DOCX) [file pone.0281196.s015.docx]

**S7 Table.**

Descriptive statistics for performance measures of the Attentional Network Test

| **Score** | **Condition** | **Study** | **Day** | **N** | **Mean** | | | **SD** | | **Min** | | **Max** | |
| --- | --- | --- | --- | --- | --- | --- | --- | --- | --- | --- | --- | --- | --- |
| Reaction Time (ms) | congruent | 1 | 1 | 15 | 540.48 | | | 64.57 | | 414.33 | | 689.35 | |
|  |  | 2 | 1 | 15 | 504.99 | | | 59.17 | | 376.35 | | 624.82 | |
|  |  |  | 2 | 15 | 472.13 | | | 46.61 | | 391.08 | | 588.23 | |
|  | neutral | 1 | 1 | 15 | 495.81 | | | 55.40 | | 381.38 | | 617.48 | |
|  |  | 2 | 1 | 15 | 469.53 | | | 55.97 | | 375.70 | | 640.27 | |
|  |  |  | 2 | 15 | 445.06 | | | 48.34 | | 363.83 | | 562.14 | |
|  | incongruent | 1 | 1 | 15 | 653.07 | | | 88.02 | | 494.79 | | 832.61 | |
|  |  | 2 | 1 | 15 | 595.60 | | | 67.66 | | 448.92 | | 750.65 | |
|  |  |  | 2 | 15 | 547.51 | | | 53.08 | | 435.04 | | 666.83 | |
|  | cue1 | 1 | 1 | 15 | 596.78 | | | 89.25 | | 445.67 | | 831.00 | |
|  |  | 2 | 1 | 15 | 555.96 | | | 80.04 | | 416.83 | | 750.65 | |
|  |  |  | 2 | 15 | 520.34 | | | 54.56 | | 417.05 | | 642.38 | |
|  | cue2 | 1 | 1 | 15 | 566.71 | | | 98.29 | | 421.50 | | 813.50 | |
|  |  | 2 | 1 | 15 | 525.74 | | | 78.96 | | 392.09 | | 706.54 | |
|  |  |  | 2 | 15 | 491.94 | | | 65.09 | | 396.26 | | 650.63 | |
|  | cue3 | 1 | 1 | 15 | 559.89 | | | 95.98 | | 415.38 | | 832.61 | |
|  |  | 2 | 1 | 15 | 525.70 | | | 79.57 | | 398.21 | | 707.39 | |
|  |  |  | 2 | 15 | 486.05 | | | 64.02 | | 384.04 | | 666.83 | |
|  | cue4 | 1 | 1 | 15 | 529.11 | | | 93.82 | | 381.38 | | 808.76 | |
|  |  | 2 | 1 | 15 | 486.07 | | | 71.19 | | 375.70 | | 687.88 | |
|  |  |  | 2 | 15 | 454.61 | | | 63.01 | | 363.83 | | 616.08 | |
| Accuracy (%) | congruent | 1 | 1 | 15 | 99.78 | | | 0.99 | | 95.24 | | 100.00 | |
|  |  | 2 | 1 | 15 | 99.79 | | | 0.94 | | 95.65 | | 100.00 | |
|  |  |  | 2 | 15 | 99.48 | | | 1.86 | | 90.91 | | 100.00 | |
|  | neutral | 1 | 1 | 15 | 99.13 | | | 2.08 | | 91.67 | | 100.00 | |
|  |  | 2 | 1 | 15 | 98.73 | | | 2.63 | | 90.91 | | 100.00 | |
|  |  |  | 2 | 15 | 99.57 | | | 1.30 | | 95.45 | | 100.00 | |
|  | incongruent | 1 | 1 | 15 | 96.14 | | | 5.91 | | 70.83 | | 100.00 | |
|  |  | 2 | 1 | 15 | 93.79 | | | 7.11 | | 65.22 | | 100.00 | |
|  |  |  | 2 | 15 | 94.15 | | | 6.30 | | 65.22 | | 100.00 | |
|  | cue1 | 1 | 1 | 15 | 97.98 | | | 3.68 | | 79.17 | | 100.00 | |
|  |  | 2 | 1 | 15 | 96.58 | | | 5.41 | | 79.17 | | 100.00 | |
|  |  |  | 2 | 15 | 97.20 | | | 3.76 | | 86.36 | | 100.00 | |
|  | cue2 | 1 | 1 | 15 | 98.29 | | | 4.23 | | 75.00 | | 100.00 | |
|  |  | 2 | 1 | 15 | 97.24 | | | 5.22 | | 77.27 | | 100.00 | |
|  |  |  | 2 | 15 | 97.42 | | | 6.32 | | 65.22 | | 100.00 | |
|  | cue3 | 1 | 1 | 15 | 97.96 | | | 5.08 | | 70.83 | | 100.00 | |
|  |  | 2 | 1 | 15 | 97.72 | | | 6.01 | | 65.22 | | 100.00 | |
|  |  |  | 2 | 15 | 97.36 | | | 4.57 | | 78.26 | | 100.00 | |
|  | cue4 | 1 | 1 | 15 | 99.17 | | | 2.45 | | 87.50 | | 100.00 | |
|  |  | 2 | 1 | 15 | 98.21 | | | 3.46 | | 86.96 | | 100.00 | |
|  |  |  | 2 | 15 | 98.96 | | | 3.06 | | 82.61 | | 100.00 | |
| Alerting | | 1 | 1 | 15 | | 36.89 | 22.43 | | 9.46 | | 93.39 | |  |
|  |  | 2 | 1 | 15 | | 30.26 | 29.17 | | -40.73 | | 73.16 | |  |
|  |  |  | 2 | 15 | | 34.29 | 15.51 | | 12.65 | | 63.33 | |  |
| Orienting | | 1 | 1 | 15 | | 37.61 | 25.48 | | -11.33 | | 83.03 | |  |
|  |  | 2 | 1 | 15 | | 39.67 | 23.95 | | -0.20 | | 87.45 | |  |
|  |  |  | 2 | 15 | | 37.34 | 18.42 | | 6.31 | | 75.89 | |  |
| Conflict | | 1 | 1 | 15 | | 112.59 | 37.80 | | 60.52 | | 202.94 | |  |
|  |  | 2 | 1 | 15 | | 90.61 | 18.62 | | 54.50 | | 121.92 | |  |
|  |  |  | 2 | 15 | | 75.38 | 17.56 | | 50.48 | | 105.90 | |  |
| Slope | | 1 | 1 | 15 | | 112.59 | 37.80 | | 60.52 | | 202.94 | |  |
|  |  | 2 | 1 | 15 | | 90.61 | 18.62 | | 54.50 | | 121.92 | |  |
|  |  |  | 2 | 15 | | 75.38 | 17.56 | | 50.48 | | 105.90 | |  |

*Note. N = sample size, SD = standard deviation, Min = minimum, Max = maximum; cue1, cue2, cue3, and cue4 represent uncued, center cued, top-bottom cued, and direction cued trials, respectively.*
