# Supplementary material for: Evaluation of the reliability and validity of computerized tests of attention
Source: PLoS One. 2023 Jan 27;18(1):e0281196. doi: 10.1371/journal.pone.0281196 (PMC9882756; doi:10.1371/journal.pone.0281196)
Supplement: S8 Table — (DOCX) [file pone.0281196.s016.docx]

**S8 Table.**

Statistics of significant main effects and interactions across Sites and post-hoc analyses

| ***Switcher*** | | | | | | | | | | |
| --- | --- | --- | --- | --- | --- | --- | --- | --- | --- | --- |
| **Standard deviation – Interaction Type x Site – F(2,96) = 4.65, η² = 0.09, p = 0.012** | | | | | | | | | | |
| Site^a^ | Type | Difference (ms) | DoF | t-value | | | Cohen’s d | | | p-value |
| BR | Type1 – Type2 | -145.78 | 96 | -3.40 | | | 0.91 | | | 0.0028 |
|  | Type1 – Type3 | -160.02 |  | -3.73 | | | 0.84 | | | 0.0009 |
| BR – CH | Type3 | 153.1 | 110 | 3.05 | | | 0.59 | | | 0.0029 |
| ***PVT*** | | | | | | | | | | |
| **Reaction time – difference across Sites** | | | | | | | | | | |
| Site | | Difference (ms) | DoF | t-value | | Cohen’s d | | | p-value | |
| CH – BR | | -75.95 | 41.1 | 5.03 | | 1.43 | | | < 0.0001 | |
| **Number of attentional lapses – difference across Sites** | | | | | | | | | | |
| Site | | Difference | W | | Cohen’s d | | | p-value | | |
| CH – BR | | -14.95 | 100 | | 1.18 | | | < 0.0001 | | |
| **Premature responses – difference across Sites** | | | | | | | | | | |
| Site | | Difference | W | | Cohen’s d | | | p-value | | |
| CH – BR | | 2.64 | 451 | | 0.56 | | | 0.0010 | | |

*Note. ^a^BR, CH = Brazilian and Swiss datasets, respectively.*
