# Supplementary material for: Evaluation of the reliability and validity of computerized tests of attention
Source: PLoS One. 2023 Jan 27;18(1):e0281196. doi: 10.1371/journal.pone.0281196 (PMC9882756; doi:10.1371/journal.pone.0281196)
Supplement: S9 Table — (DOCX) [file pone.0281196.s017.docx]

**S9 Table.**

Significant correlations between task scores and questionnaire scores

| **DSSQ** | | | |
| --- | --- | --- | --- |
| **Questionnaire score** | **Psychological task score** | **Spearman's rank correlation coefficient (ρ)** | **Unc. p-value** |
| Success motivation | PVT slope | -0.56 | 0.004* |
|  | ANT orientation | 0.43 | 0.03 |
| Energic arousal | CPT RT ISI2 | -0.41 | 0.04 |
| Task-relevant interference | CPT RT ISI2 | 0.43 | 0.03 |
|  | ANT RT congruent | 0.42 | 0.04 |
| Intrinsic motivation | PVT RT | -0.48 | 0.015 |
|  | PVT slope | -0.41 | 0.04 |
|  | ANT orientation | 0.46 | 0.02 |
| Concentration | Rotation RT 135° | 0.48 | 0.014 |
| Control and confidence | ANT RT congruent | 0.40 | 0.05 |
|  | ANT RT incongruent | 0.42 | 0.04 |
| Task-irrelevant interference | ANT RT congruent | 0.42 | 0.05 |
|  | ANT RT incongruent | 0.43 | 0.03 |
| Anger/frustration | ANT slope | 0.46 | 0.021 |
| **CFQ** | | | |
| CFQ | CPT RT ISI1 | -0.66 | 0.03 |
|  | Rotation RT 0° | -0.74 | 0.012 |
|  | Rotation RT 135° | -0.62 | 0.05 |
|  | ANT slope | 0.69 | 0.023 |

*Note. Uncorrected p-values < 0.05. The asterisk indicates significance after correction for multiple comparisons using the FDR method. The attention tasks are represented with italic letters and further information about the condition in which the significant differences were found and the F-test statistics (when applicable) are represented with bold letters.*
