# Supplementary material for: Evaluation of the reliability and validity of computerized tests of attention
Source: PLoS One. 2023 Jan 27;18(1):e0281196. doi: 10.1371/journal.pone.0281196 (PMC9882756; doi:10.1371/journal.pone.0281196)
Supplement: Attachment — Submitted filename: Response_to_reviews_Langner2023_PlosOne2.docx [file pone.0281196.s019.docx]

Zurich, 17^th^ January 2023

**Revision of manuscript PONE-D-22-16441R1**

*Evaluation of the reliability and validity of computerized tests of attention*

Dear Dr Males, dear Dr. Fernández-Marcos,

We would like to thank you again for the new review. Please find our responses to your comments below, which we have considered and further improved the manuscript. For your convenience, as in the first response letter, we have used bold font to denote the reviewer's comments, and blue/italic font to highlight new text. All line numbers refer to the “Revised manuscript with tracked changes”.

We appreciate your attentive reading and the new suggestions and hope that you will find the revised version acceptable for publication in PLoS One.

Sincerely, on behalf of all authors,

Gustavo Pamplona, PhD

--

Postdoctoral Fellow

Department of Ophthalmology, Hôpital Ophtalmique Jules-Gonin

University of Lausanne

Avenue de France 15

1004 Lausanne

Tel: +41 76 281 88 87

gsppamplona@gmail.com

**Response to Reviews**

# 1. Reviewer 2

**The authors present a study of the validity and reliability of 5 widely used computerized neuropsychological tests included in an open-source software program (PEBL, Psychology Experiment Building Language).**

**While this is an interesting tool and computerized neuropsychological assessment is gaining popularity since combining computerized tools and standard neuropsychological tests seems to provide the greatest value in clinical assessment, the sample size is an important limitation to draw firm conclusions but still the authors made a useful starting point.**

**Specific considerations are listed below:**

## 1.1. Comment 1

**In the second paragraph in the ‘Introduction’ section, I suggest to briefly mention the most appropriate statistical methods used to measure reliability and validity constructs.**

We have inserted the statistical methods in lines 59-60 and 65 as suggested by the reviewer.

## 1.2. Comment 2

**In line 71 the limited evidence of the PEBL should be described in depth with the references and limitations of these studies.**

Although some studies were conducted to assess the psychometric properties of PEBL tasks, they usually did it for individual studies (therefore, associations between PEBL tasks or with cognitive questionnaires are rare), there is no evidence of validity or reliability for some tasks (particularly for the Switcher test), and we are not aware of studies on the stability across countries using the PEBL tests. We edited the text between lines 70 and 78 to make this fact explicit:

*“Among other cognitive functions, PEBL enables the assessment of various aspects of attention via a range of tasks. Although widely utilized [3], there is currently limited evidence on the psychometric properties of the available tasks in PEBL [3,12–17]. Such studies, altogether, show only fragmented evidence of the psychometric properties across PEBL tasks dedicated to measuring attention. In fact, evidence on reliability or validity is entirely lacking for certain PEBL tasks (for example, the Switcher task), an evaluation across sites of application has never been done, and data on relationships between PEBL’s attentional measures and cognitive questionnaires are scarce. Thus, evaluating the reliability and validity of PEBL tasks measuring aspects of attention is an important issue.”*

## 1.3. Comment 3

**In line 73 ‘Among other mental functions, PEBL enables the assessment of various…’ I recommend to better use cognitive functions instead of mental functions.**

We thank the reviewer for this pointer. We have corrected the indicated sentence (line 70).

## 1.4. Comment 4

**The description of the tasks (line 74 to 92) should be included in the Methods Section (Experimental procedures)**

We thank the reviewer for the suggestion. However, we think that first introducing the tasks whose psychometric properties will later be evaluated would be beneficial for this manuscript. Although the focal point of the manuscript is the psychometric properties of the tasks, rather than the tasks per se, we think it is critically important that the reader knows from the outset about the tasks to be assessed.

This way, we aimed to separate between *what* the task measures (which aspect of attention) and *how* the task accomplishes it (the experimental procedures). Such distinction was made by describing the former in the Introduction and the latter in Methods – Experimental procedures and measures.

## 1.5. Comment 5

**I suggest the authors to add to the section Experimental procedures ‘and measures’. For improving the value of this study, I suggest organizing this section:**

**- First part, paragraph 1 (line 139 to 147)**

**- Second part, paragraph 3 (line 168 to 186)**

**- Third part would include the procedure (a graphical explanation of the study design would help) and the description of the tasks including the measures that were selected for this study (Table 2).**

We edited the manuscript as suggested by the reviewer. We agree that reordering the paragraphs made the section clearer. We also included a graphical representation of the timeline for each study, as suggested by the reviewer (Fig. 1A). Because now Figure 1 contains two subdivisions (A and B), we included references to the subdivisions of the figure whenever appropriate (lines 153, 163, 165, 170, and 187). We also edited the caption from Figure 1 to describe the new graphical information (line 196):

*“(A) Timeline of the studies. Upon receiving the instructions, participants completed the questionnaires (the Dundee Stress State Questionnaire (DSSQ) and the Cognitive Failures Questionnaire (CFQ) for Study I, only the DSSQ for Study II, and only the Brunel Mood Scale (BRUMS) validated to Brazilian-Portuguese for Study III). Next, participants performed the computerized tests, always in the same order (Continuous Performance Task (CPT), Switcher Task, Psychomotor Vigilance Task (PVT), Mental Rotation, and Attentional Network Test (ANT) for Studies I and II, and CPT, Switcher, PVT, and Stroop Task for Study III. The results for the Stroop Task were not analyzed in this study. Participants took five-minute breaks after conducting two tasks. (B) Screenshots of representative conditions and instructions for the PEBL attention tasks used. Tasks are color-coded as in A.”*

## 1.6. Comment 6

**The section Data analysis includes repeated information (line 279 to 281).**

We removed the following excerpt (line 279), avoiding repeating the information previously given:

*“: data from CPT, Switcher Task, and PVT were available for all Studies; data from Mental Rotation task and ANT were only available for Studies I and II”*

## 1.7. Comment 7

**Although the PEBL (and e.g. in line 700 in this study) uses the term psychological tasks to refer to the CPT, Digits Span and so on, I would consider using a more specific term such as attentional, cognitive or neuropsychological tasks since it could be confusing.**

In order to avoid this lack of specificity pointed out by the reviewer, we have changed our wording from “psychological” tasks to either “cognitive” (lines 70, 703) or “attentional” tasks (lines 28, 51 and 764) in several instances of the text.
